# Distribution, abundance, and ecogenomics of the *Palauibacterales*, a new cosmopolitan thiamine-producing order within the *Gemmatimonadota* phylum

**DOI:** 10.1128/msystems.00215-23

**Published:** 2023-06-22

**Authors:** Borja Aldeguer-Riquelme, Josefa Antón, Fernando Santos

**Affiliations:** 1 Department of Physiology, Genetics, and Microbiology, University of Alicante, Alicante, Spain; 2 Multidisciplinary Institute of Environmental Studies Ramón Margalef, University of Alicante, Alicante, Spain; Oregon State University, Corvallis, Oregon, USA

**Keywords:** *Gemmatimonadota*, *Palauibacterales*, PAUC43f, KS3-K002, cosmopolitan, thiamine, marine, microbial ecology

## Abstract

The phylum *Gemmatimonadota* comprises mainly uncultured microorganisms that inhabit different environments such as soils, freshwater lakes, marine sediments, sponges, or corals. Based on 16S rRNA gene studies, the group PAUC43f is one of the most frequently retrieved *Gemmatimonadota* in marine samples. However, its physiology and ecological roles are completely unknown since, to date, not a single PAUC43f isolate or metagenome-assembled genome (MAG) has been characterized. Here, we carried out a broad study of the distribution, abundance, ecotaxonomy, and metabolism of PAUC43f, for which we propose the name of *Palauibacterales*. This group was detected in 4,965 16S rRNA gene amplicon datasets, mainly from marine sediments, sponges, corals, soils, and lakes, reaching up to 34.3% relative abundance, which highlights its cosmopolitan character, mainly salt-related. The potential metabolic capabilities inferred from 52 *Palauibacterales* MAGs recovered from marine sediments, sponges, and saline soils suggested a facultative aerobic and chemoorganotrophic metabolism, although some members may also oxidize hydrogen. Some *Palauibacterales* species might also play an environmental role as N_2_O consumers as well as suppliers of serine and thiamine. When compared to the rest of the *Gemmatimonadota* phylum, the biosynthesis of thiamine was one of the key features of the *Palauibacterales*. Finally, we show that polysaccharide utilization loci (PUL) are widely distributed within the *Gemmatimonadota* so that they are not restricted to *Bacteroidetes*, as previously thought. Our results expand the knowledge about this cryptic phylum and provide new insights into the ecological roles of the *Gemmatimonadota* in the environment.

**IMPORTANCE:**

Despite advances in molecular and sequencing techniques, there is still a plethora of unknown microorganisms with a relevant ecological role. In the last years, the mostly uncultured *Gemmatimonadota* phylum is attracting scientific interest because of its widespread distribution and abundance, but very little is known about its ecological role in the marine ecosystem. Here we analyze the global distribution and potential metabolism of the marine *Gemmatimonadota* group PAUC43f, for which we propose the name of *Palauibacterales* order. This group presents a saline-related character and a chemoorganoheterotrophic and facultatively aerobic metabolism, although some species might oxidize H_2_. Given that *Palauibacterales* is potentially able to synthesize thiamine, whose auxotrophy is the second most common in the marine environment, we propose *Palauibacterales* as a key thiamine supplier to the marine communities. This finding suggests that *Gemmatimonadota* could have a more relevant role in the marine environment than previously thought.

## INTRODUCTION

Over the last three decades, the development of culture-independent techniques has allowed the study of many microbial taxa that had remained hidden due to culture limitations. Among these taxa, the phylum *Gemmatimonadota* was discovered in 2001 by two independent studies that used 16S rRNA gene clone libraries to explore the microbial diversity of a reactor sludge and coastal marine sediments (1, 2). Formerly designated as “candidate division BD” (or KS-B), this phylum was renamed in 2003 when the strain T-27^T^ was isolated from a wastewater treatment plant and named *Gemmatimonas aurantiaca* (3). The phylum contains seven classes based on 16S rRNA gene phylogeny (*Gemmatimonadetes*, *Longimicrobia*, PAUC43f marine benthic group, BD2-11 terrestrial group, S0134 terrestrial group, AKAU4049, and MD2902-B12), but only the *Gemmatimonadetes* and *Longimicrobia* have cultured representatives. In fact, approximately 86% of all 16S rRNA gene sequences of *Gemmatimonadota* deposited in the SILVA database have been retrieved from uncultured members of the phylum.

Previous studies based on 16S rRNA gene sequences have highlighted *Gemmatimonadota* as a cosmopolitan phylum, as diverse as *Actinobacteria* or *Proteobacteria* (4), which maybe indicating a broad physiological diversity allowing this group to colonize a great variety of environments. Accordingly, G*emmatimonadota* are present in many types of soils, where they constitute one of the eight most abundant phyla, accounting for up to 6.5% of total 16S rRNA gene sequences (5, 6). Recently, Bay and coworkers suggested the metabolic potential of soil *Gemmatimonadota* MAGs to oxidize CH_4_ and reduce N_2_O, both potent greenhouse gases (7). Indeed, *in vitro* experiments with *G. aurantiaca* had previously confirmed its ability to reduce N_2_O (8, 9). *Gemmatimonadota* are also present in the water column and sediments of freshwater lakes (10 - 13). These environments harbor both chemoorganotrophic and photoheterotrophic *Gemmatimonadota*, as revealed by cultures (14, 15) and metagenomics (10, 11). A recent study in Czechia and Switzerland’s freshwater lakes estimated that *Gemmatimonadota* could represent up to 1% of the planktonic microbial community, with the highest relative abundances in the hypolimnion (11). Finally, *Gemmatimonadota* have also been found in marine environments, such as seawater (16, 17), marine sediments (18 - 21), and sponges (4, 22, 23). Due to this ubiquity in marine environments, Hanada and Sekiguchi, in 2014, suggested that *Gemmatimonadota* may play an important role, albeit still unexplored, in the oceans (4).

PAUC43f is one of the most frequently detected classes of *Gemmatimonadota* in marine environments (4). However, although the first 16S rRNA gene sequence assigned to this class was discovered 20 yr ago (24, 25), and it is the third largest class of *Gemmatimonadota* in the SILVA database, very little is known about its ecology and physiology. Indeed, to date, PAUC43f members have been detected only through 16S rRNA gene sequences, and there is not a single isolate or metagenome-assembled genome (MAG) affiliated with this group. Published data suggest that PAUC43f members are salt-adapted, present in marine sediments, hydrothermal vents, sponges, and corals (19, 26 - 31) and also in ephemeral saline lake sediments (32, 33), although their phylogenetic breadth, metabolic potential, and ecological role remain unexplored.

In this work, we aim to fill the gap of information about the distribution, abundance, physiology, and ecological role of the *Gemmatimonadota* PAUC43f group. For this purpose, we retrieved all the PAUC43f 16S rRNA gene sequences from SILVA r138 and performed an extensive search for the group in 189,104 publicly available 16S rRNA gene amplicon datasets from the Sequence Read Archive (SRA). Several databases were also screened for PAUC43f MAGs that had been previously overlooked. Our results confirmed the widespread distribution of PAUC43f in salt-related environments (brackish to hypersaline, with the highest abundances in sponges and marine sediments) and also in soils. Based on 16S rRNA gene sequences, 16 new genera were defined and linked to different ecological niches. The characterization of the metabolic potential of some members of PAUC43f indicated that they may reduce N_2_O and thus be helpful for mitigating the harmful effects of this potent greenhouse gas. In addition, the potential capability to synthesize serine and vitamin B1 (thiamine) was found in most PAUC43f MAGs, suggesting that they might play an important role by supplying these compounds to the community.

## MATERIALS AND METHODS

### PAUC43f 16S rRNA gene analyses

**Fig 1:**
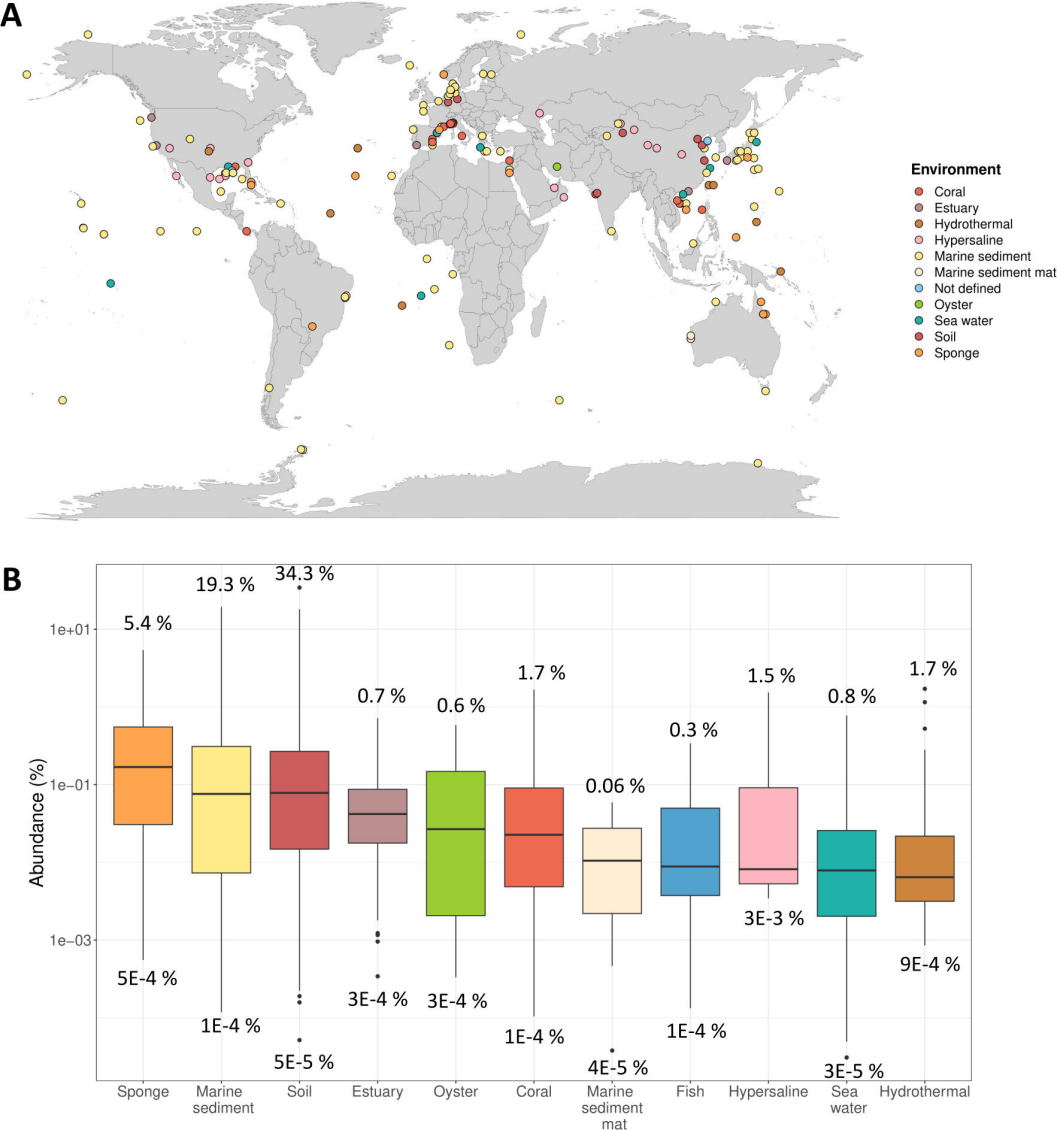
Ecological distribution and abundance of PAUC43f based on 16S rRNA gene sequences. (**A**) Worldwide distribution and environments where PAUC43f sequences have been detected. (**B**) Boxplot, in logarithmic scale, of PAUC43f relative abundances in different environments, measured as the percentage of PAUC43f 16S rRNA gene sequences with respect to the total number of 16S sequences (see *P*-values for Wilcoxon pairwise comparisons in Table S2). Colors correspond to the sampled environment. Values above and below each boxplot indicate the maximum and minimum abundance, respectively, in each environment. Number of datasets per environment: sponge, *n* = 324; marine sediment, *n* = 1,997; soil, *n* = 1,049; estuary, *n* = 295; oyster, *n* = 63; coral, *n* = 195; marine sediment mat, *n* = 28; fish, *n* = 41; hypersaline, *n* = 6; seawater, *n* = 930; and hydrothermal, *n* = 30.

A dataset was built with complete and partial 16S rRNA gene sequences, classified as “PAUC43f marine benthic group” retrieved from SILVA r138 database (34), marine invertebrates (corals and sponges from the Mediterranean Sea (35)), and sediments from the Mar Menor lagoon (SE, Spain (36)). For the following bioinformatic analyses, default settings were used unless otherwise noted. Sequences were aligned using SINA (37), in the ARB software (38), and introduced by parsimony into the SILVA 16S rRNA tree to check their taxonomy. Only 3,686 sequences, clustering within the PAUC43f group, were kept for further analyses. To avoid redundancy, sequences were clustered with cd-hit-est v4.8.1 (39) at 97% of identity, a threshold commonly used for species delineation (40), and 90% of coverage (-c 0.95 -aS 0.9). As a result, 384 groups were generated, and the longest sequence of each group was selected as the representative for subsequent analyses. The map with the global distribution of PAUC43f (Fig. 1A) was drawn in R with the ggplot2 v3.3.5 (41) and tidyverse v1.3.1 (42) packages, based on the type of environment and geographic coordinates provided in the 179 sequences, out of the 384 representatives, for which metadata were available.

The presence of PAUC43f in different environments was estimated using the IMNGS software (43). The abovementioned 384 representative sequences were searched in a total of 189,104 16S rRNA gene amplicon datasets, available in the SRA repository, from 16 different environments (air, coral, estuary, fish, freshwater, human gut, human not gut, hydrothermal, hypersaline, marine sediment, marine sediment mat, seawater, oyster, skin, soil, and sponge) using a 97% identity cutoff. To obtain a more precise value of PAUC43f relative abundances, estimated by the percentage of total 16S rRNA gene sequences, those SRA datasets where PAUC43f was detected by IMNGS (4,965 datasets corresponding to 11 environments) were downloaded, BLASTN-queried (-outfmt “6 qseqid sseqid pident length mismatch gapopen qstart qend sstart send evalue bitscore qlen slen”) against the 384 representative sequences, and only best hits (BlastTab.best_hit_sorted.pl) above 97% identity and 70% coverage were considered (awk “{if($4/$13>=0.7 && $3>=97)print$0}”). Since the sequences not meeting these criteria were not used for calculating PAUC43f relative abundance, the calculated values likely underestimate the true abundance of this group in the abovementioned environments.

For precise taxonomic studies, the 66 sequences longer than 800 bp (from the 384 representative sequences) were analyzed in the ARB software v6.0.6. SINA was used to align the sequences and, to exclude highly variable positions, a base frequency filter was applied prior to the tree construction. First, the tree was constructed with the 45 sequences longer than 1,200 bp with both neighbor-joining (Jukes-Cantor correction) and maximum likelihood (PHYML) algorithms (1,000 bootstraps). Then, 21 sequences, between 800 and 1,200 bp, were added by parsimony. Sequences from classes BD2-11, MD2902-B12, and *Gemmatimonadetes* were used as outgroups. A cluster representing a genus was defined when at least two sequences were monophyletic in both neighbor-joining and maximum likelihood trees (44, 45) and their identities were above 94.5% of the threshold for genus delineation (46). Finally, iTOL was employed to draw the tree (47). The environmental frequency and abundance of each genus were estimated as explained above for the 384 representative sequences. The frequency of each genus in each environment was defined as the number of samples where the genus was detected with respect to the total number of samples with presence of PAUC43f.

### Metagenome-assembled genome analyses

MAGs belonging to *Gemmatimonadota* were searched in the GTDB release 207 (48) and GEM databases (49) as well as in other public sources (https://data.ace.uq.edu.au/public/sponge_mags/; (22, 23, 50)) and Mar Menor sediments samples described in (36). DNA from Mar Menor sediments (stations 2, 3, 5, 10, 13, 20, and 21) was extracted with the DNeasy PowerSoil kit (Qiagen) following the manufacturer’s indications, and metagenomes were sequenced on an Illumina Novaseq 6000 2×150 bp run in the CNAG (Barcelona, Spain). Raw reads were quality filtered and adapters removed using Trimmomatic v0.36 (LEADING:3 TRAILING:3 SLIDINGWINDOW:4:15 MINLEN:36) (51) and then, megahit v1.2.9 (52) was used to assemble reads. Then, contigs (>2 Kb) were binned using MaxBin2 v2.2.7 (53) and MetaBAT2 v2.15 (54) and, finally MAGs were refined with DAS_Tool v1.1.3 (55).

To identify PAUC43f MAGs from the pool of *Gemmatimonadota* genomes and MAGs, 16S rRNA gene sequences were extracted and classified in the online SILVA ACT service (https://www.arb-silva.de/aligner/). MAGs carrying a 16S rRNA gene sequence of PAUC43f were classified, using the whole genome classifier tool GTDB-tk v2.1.1 r207 (classify_wf) (48), within the order KS3-K002, in the *Gemmatimonadetes* class. For this reason, all *Gemmatimonadota* MAGs lacking a 16S rRNA gene sequence but assigned to order KS3-K002 were considered as PAUC43f.

MAGs considered as PAUC43f were manually curated by removing contigs with different sequencing depths or incongruent taxonomic affiliation of proteins, as previously proposed (56). To calculate sequencing depth, the metagenomic reads were mapped against the MAGs by BLASTn (-outfmt “6 qseqid sseqid pident length mismatch gapopen qstart qend sstart send evalue bitscore qlen slen”), hits were filtered by best hit, 70% coverage breadth, and 95% identity, and finally the BlastTab.seqdepth.pl script of Enveomics calculated the sequencing depth values. Contigs with more than twofold change with respect to the mean sequencing depth were removed. Regarding the taxonomic affiliation, MAG’s proteins were queried against the nr database using DIAMOND BLASTp v0.9.21.122 (-outfmt “6 qseqid sseqid pident length mismatch gapopen qstart qend sstart send evalue bitscore qlen slen stitle”), retaining only the best hit for each protein. The most common taxonomic classification was determined by visual inspection, and contigs that do not present proteins classified to these taxa were removed. Completeness and contamination were estimated using CheckM 1.1.3 (lineage_wf) (57). To calculate the estimated genome size, MAG assembly size was divided by CheckM completeness (ranging from 0 to 1) (58). ANOVA was used to test for statistically significant differences in genome size with regard to the origin using the aov function (R stats) and the HSD.test function of the agricolae package (unbalanced=TRUE, group=FALSE) (59). Phylogenomic tree for the 441 genomes and MAGs classified as *Gemmatimonadota,* which includes the orders *Gemmatimonadales*, *Longimicrobiales*, PAUC43f (=*Palauibacterales*), JACCXV01, and the classes *Glassbacteria* and GCA-2686955, was then constructed with PhyloPhlAn v3.0.58 (60) using *Robiginitalea biformata* HTCC2501 as outgroup. Phylogeny was inferred from the alignment of 400 marker genes by the RAxML maximum-likelihood algorithm. To calculate MAG abundances, metagenomic reads were mapped by BLASTn (-outfmt “6 qseqid sseqid pident length mismatch gapopen qstart qend sstart send evalue bitscore qlen slen”), hits were filtered by best hit using the enveomics script BlastTab.best_hit_sorted.pl, coverage > 70%, and identity > 95% (awk “{if($4/$13>=0.7 && $3=95)print$0}”). Normalized abundance was calculated as the number of mapped reads divided by metagenome and genome size. Metabolic reconstruction was carried out using the annotation provided by KofamScan v1.3.0 (61) and Interproscan v5.57–90.0 (-appl CDD, Pfam, SMART, TIGRFAM) (62 - 66). Secondary metabolite biosynthetic gene clusters (BGCs) were identified by antiSMASH v6.1.1 (67) with the “strict” detection level. CAZymes were annotated against dbCAN V11 (68) by DIAMOND BLASTp v0.9.21.122 (identity > 40%, coverage > 50%) and HMMER v3.3.1 (e-value < 1e-15, coverage > 0.35) (69), considering only hits reported by both strategies. Statistically significant differences in abundance among environments and between the number of CAZymes and susCD genes per order were tested in R using the Kruskal-Wallis (kruskal.test) and Wilcoxon (pairwise.wilcox.test) tests. For the latter, p-values were corrected by Bonferroni afnd, to avoid biased results due to small group size, only groups with more than 16 samples were evaluated, as previously indicated (70).

To identify genomic and metabolic differences between the *Gemmatimonadales*, *Longimicrobiales,* and *Palauibacterales* orders, the 415 available *Gemmatimonadota* genomes and MAGs were dereplicated at 95% ANI (species threshold (71)) using dRep v3.2.2 (72), and only those with completeness above 80% and contamination below 5% were considered (dereplicate -comp 80 -con 5 -sa 0.95). As a result, 215 genomes and MAGs were obtained and analyzed by Anvi’o 7.1 to get the enriched KEGG modules (-module-completion-threshold 0.8 and qvalue < 0.01) (73).

### Fluorescence *in situ* hybridization (FISH)

To get experimental information (presence, activity, morphology, and size) about PAUC43f, PCR primers and FISH probes were designed using DECIPHER (74) and PrimerQuest Design Tool (IDT, https://eu.idtdna.com/PrimerQuest/Home/Index). Since Mar Menor sediment samples (Murcia, Spain; 37°45'N 0°47'W), where PAUC43f had been previously detected, were readily accessible to our lab, we designed primers and probes against the 16S rRNA gene sequences of PAUC43f retrieved from these sediments (36). *In silico* quality control was performed using the OligoAnalyzer Tool (IDT, https://eu.idtdna.com/pages/tools/oligoanalyzer), searching for secondary structures and dimerization, while probe specificity was checked with TestProbe against the SILVA database (34). As a result, the probe PAUC43f_826 (5′- AGGGTCAATCCTCCCAACACCTAGTAC-3′), which covered 32.7% of the PAUC43f sequences from SILVA, was selected as the best candidate. To test the probe, a sediment sample from the Mar Menor lagoon (37°40'02.8"N 0°48'55.2"W) was collected in the summer of 2021 and fixed with 4% formaldehyde at 4°C for 4 h. Before hybridization, the presence of PAUC43f in these samples was confirmed by PCR with specific primers for this group (272F: 5′-GTAAGTCGGGTGTGAAATTC-3′; and 393R: 5′-TTCCCGATATCTACGCATTC-3′) which covered 11.2% of SILVA’s PAUC43f sequences. The hybridization was carried out on a filter, as previously described (75), and the probe was optimized using six different formamide concentrations (10%, 20%, 30%, 40%, 50%, and 60%). Briefly, hybridization was done at 46°C for 4 h, followed by two washing steps at 48°C for 15 min. Then, filters were stained with DAPI (1 mg/mL), washed with milli-Q water, dehydrated with absolute ethanol (1 min each step), and finally visualized in the Zeiss LSM800 confocal laser scanning microscope.

## RESULTS AND DISCUSSION

### Ecological distribution of PAUC43f

PAUC43f 16S rRNA gene sequences were detected in several marine environments (such as sediments, sediment mats, corals, sponges, oysters, estuaries, seawater, and hydrothermal vents), hypersaline lake sediments, and soils (Fig. 1A). A large proportion of the sequences (89 out of 179) were recovered from marine sediment samples. Regarding geographical distribution, PAUC43f has been detected around the world in almost every latitude and longitude, and in both shallow and deep aquatic environments.

To get more insights into the PAUC43f ecological distribution, its relative abundance (as a percentage of PAUC43f 16S reads from the total 16S reads) was estimated for each environment (Fig. 1B). PAUC43f was detected in 4,965 of the 189,104 16S rRNA gene amplicon datasets analyzed, mainly from the marine environment, supporting the definition of PAUC43f as an essentially “salt-related” group (see Table S1 for the available salinity values). The group is also present in soils, some of them saline. However, since metadata for most soil samples were not available, the presence of PAUC43f in non-saline soils cannot be ruled out. The highest mean relative abundances were in sponges, marine sediments, and soils, while the lowest values were found in seawater and hydrothermal vent samples (Fig. 1B; Table S2). The extremely high relative abundance of PAUC43f in some samples is remarkable, such as an arid saline soil in China (76) and petroleum-impacted sediments from a saline lake in the Egyptian Red Sea (77), reaching up to 34.3% and 19.3%, respectively.

Since PAUC43f reached significantly higher relative abundances in sponges, marine sediments, and soils (Table S2), its distribution in these environments was explored more deeply. PAUC43f was detected in at least 30 different sponge species, found most frequently in *Coscinoderma matthewsi* (87 samples, where it accounted for up to 5.4% of the 16S rRNA gene sequences), *Xestospongia* spp. (71 samples), *Rhopaloeides odorabile* (32 samples), and *Suberites* spp (16 samples). Regarding marine sediments, no clear pattern of distribution was observed in relation to latitude, water temperature, or water column depth above the sediment (Fig. S1A through C). However, the depth along the sediment did seem to be important since PAUC43f abundances were highest at the surface and they decreased with depth (Fig. S1D). For soils, the highest abundances were found in middle latitudes in the Northern Hemisphere (Fig. S2A), although it must be noted that this hemisphere presents a higher proportion of land than the Southern Hemisphere. As for sediments, the abundance of PAUC43f in soils was also higher at the surface (Fig. S2B). These observations were not influenced by the different number of samples available for each depth (Fig. S3).

### Ecotaxonomy

**Fig 2:**
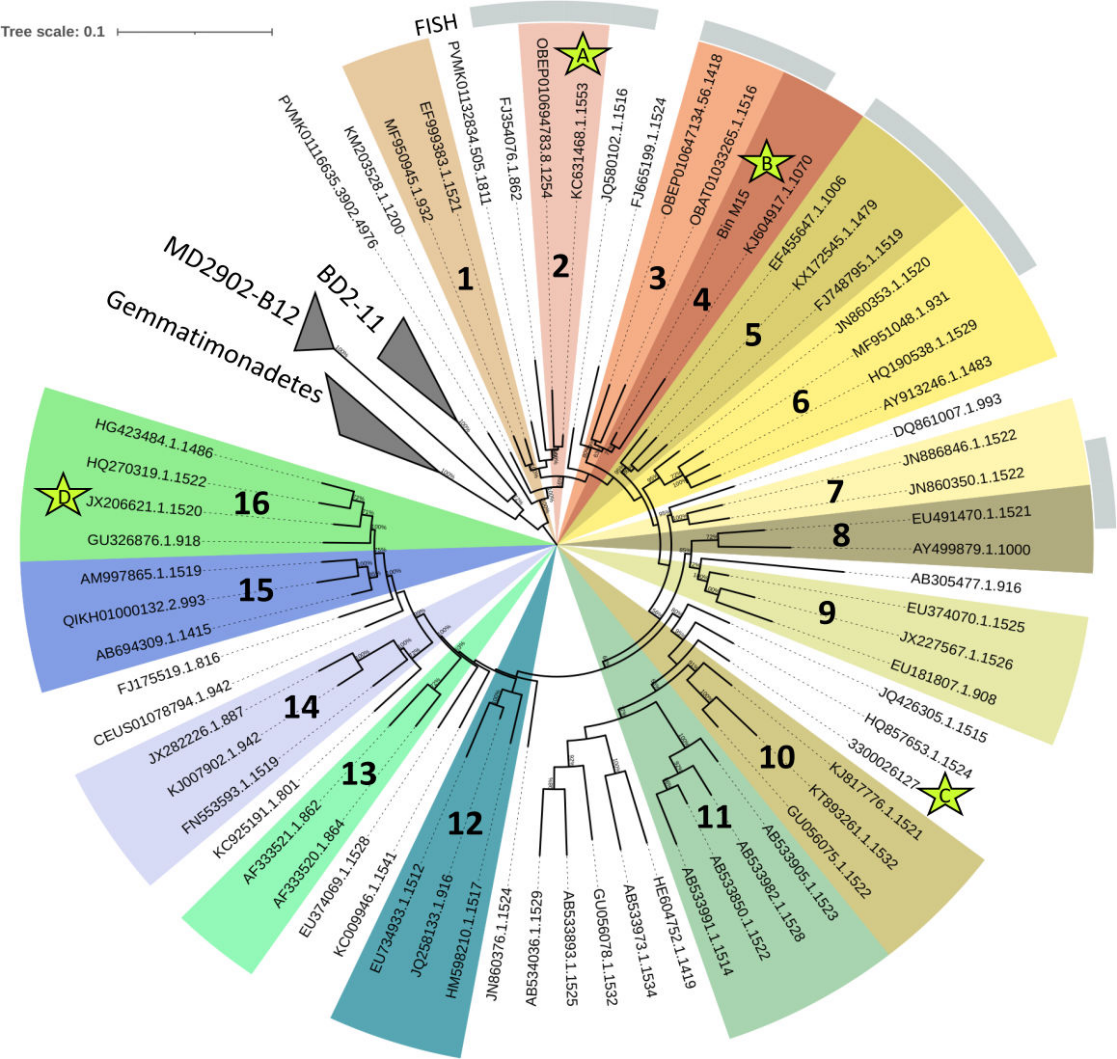
Maximum-likelihood tree based on 66 PAUC43f 16S rRNA gene sequences longer than 800 bp. Monophyletic clusters in both NJ and maximum-likelihood trees with identities above 94.5%, the threshold for delineating genera, are displayed with different colors and numbers. The external grey circle indicates the sequences targeted by the FISH probe. The 16S rRNA gene sequences from MAGs are marked with stars. Star A: 3300025554_5; Star B: Bin_M15_27; Star C: 3300026127_2; and Star D: RHO2_bin_49.

The 16S-based phylogenetic tree revealed 16 PAUC43f genera, supported by both neighbor-joining and PHYML algorithms (Fig. 2), which included 62% of the total tree sequences. These genera, altogether with the rest of the sequences included in the tree (except AB305477.1.916), belong to the same order and the same family, based on previously proposed thresholds for these taxonomic ranks (82.0% and 86.5% identity of 16S rRNA gene sequence, respectively (46)).

**Fig 3:**
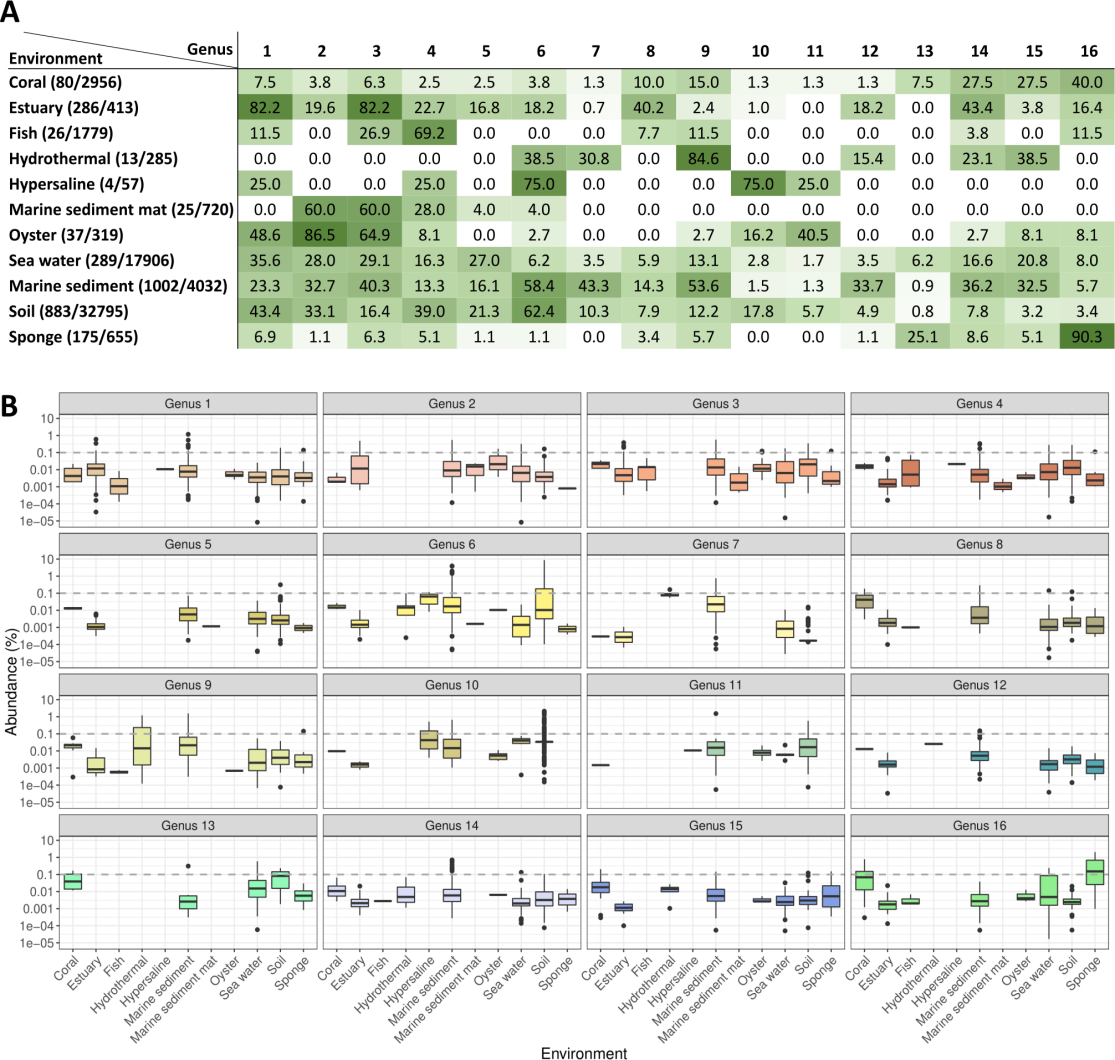
Ecological distribution of 16 PAUC43f genera. (**A**) Percentage of samples per environment where each genus is detected with respect to the total number of samples where PAUC43f is present. Highest values are displayed in green and lowest in white. Beside each environment name, in parentheses, there are the numbers of samples where PAUC43f genera were detected out of the total number of samples analyzed for that environment. (**B**) Relative abundance of each genus in each environment, as percentage of PAUC43f 16S rRNA gene sequences with respect to the total number of 16S sequences. The horizontal dashed line indicates a relative abundance of 0.1%, as a threshold for abundant and rare biospheres. (see p-values for Wilcoxon pairwise comparisons in Table S2).

To analyze the ecological distribution of these genera, their frequencies and abundances in different environments were calculated. As shown in Fig. 3A, the detection frequency of each genus differed across environments. Some genera, such as 1, 3, 4, 6, and 9, were generalists, displaying a wide environmental distribution, while others, such as genera 10, 11, 12, and 13, were more limited to a few environments and samples. All genera were detected in corals, seawater, marine sediments, and soils, whereas only a few were found in fish, hydrothermal vents, hypersaline lake sediments, and marine sediment mats. According to their relative abundances (Fig. 3B), PAUC43f genera might be included in the rare biosphere of many environments (relative abundances <0.1% (78)). However, in certain samples, some genera showed moderate to high relative abundances (>0.1%). For example, genera 6 and 10 were significantly more abundant in marine sediments and soils than in the other samples (Table S2). Genus 16, mostly host-associated, had significantly higher abundances in corals and sponges (Table S2), and genera 7 and 9 displayed abundances above 0.1% in hydrothermal vents and marine sediments. These observations suggest that each genus might be better adapted to specific environments, which implies that at least some genera could be genuine members of microbiomes of corals, sponges, marine sediments, hypersaline lake sediments, and soils. Other genera, due to their low abundances and frequencies in fish, marine sediment mats, and oysters, might likely be transient inhabitants of these environments.

### Phylogenomic analyses and description of order *Palauibacterales*

The search of genomes/MAGs in databases (GEM & GTDB r207) and recent publications (22, 23, 50) led to the identification of 37 PAUC43f MAGs: 19 from GTDB, 8 from the GEM database, and 10 from recent publications. Fifteen additional MAGs were recovered from Mar Menor sediments (see Methods). Out of these 52 PAUC43f MAGs (Table S3), 45 could be considered of good quality according to the published criteria (completeness above 80% and contamination below 5% [56, 71]); 15 of them also carried 16S rRNA genes (Table S3). The estimated MAG sizes ranged from 1.9 to 4.3 Mb, with GC contents between 52.8% and 71.7%. Regarding their origins, the MAGs were obtained from marine sediments, sponges, saline soils, sea water and ground waters (26, 21, 3, 1, and 1 MAGs, respectively, Suppl. Table 3). A statistically supported relationship between MAG origin and estimated genome size was observed, with the smallest genomes found in marine sediments and the largest in sponges (Suppl. Figure 4), independently of their completeness. In terms of relative abundance, most MAGs accounted for more than 0.1% (and up to 12.52%, Suppl. Table 3) of total reads in their original metagenomes and thus, belonged to the abundant biosphere.

**Fig 4:**
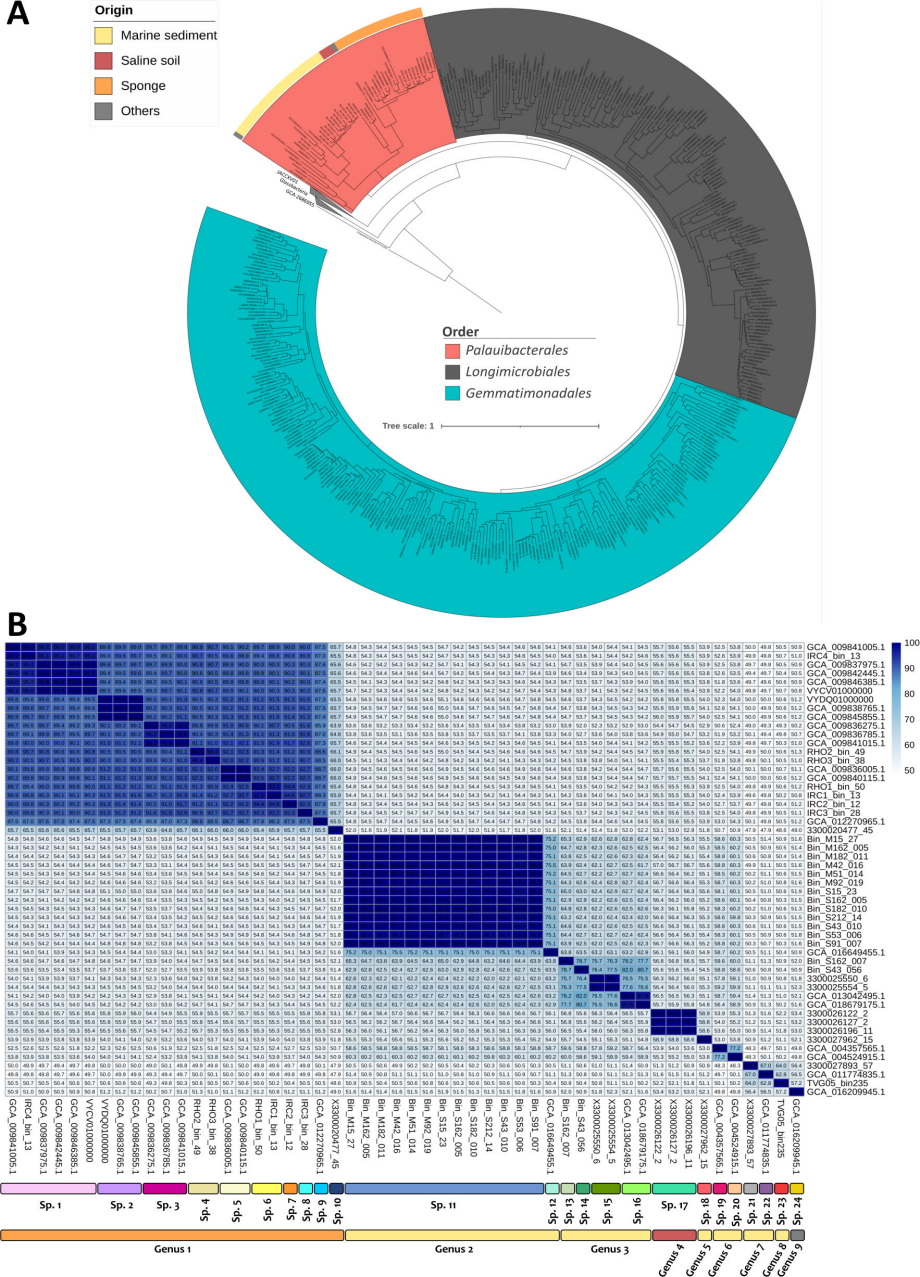
Taxonomic classification of *Palauibacterales* MAGs. (**A**) Phylogenomic tree with all available *Gemmatimonadota* MAGs. In *Palauibacterales*, the external circle indicates the environment where the MAG was recovered. The genome of *Robiginitalea biformata* HTCC2501 was used to root the tree. (**B**) Heatmap based on average amino acid identity (AAI) values for the *Palauibacterales* MAGs. Values above 95% of AAI, the threshold for species delimitation, are highlighted in dark blue. At the bottom, genera colors indicate the environment where the MAG was recovered following the same color schema as in A.

A phylogenomic tree using all available *Gemmatimonadota* genomes and MAGs supported the monophyletic origin of PAUC43f within this phylum. Contrary to the SILVA and in agreement with GTDB classification, PAUC43f (=KS3-K002) is likely a new order within class *Gemmatimonadetes* rather than a new *Gemmatimonadota* class (Fig. 4A). Within the order, PAUC43f MAGs recovered from marine sediments, sponges, and saline soils clustered in three different subbranches, respectively. A similar result was obtained when the AAI among these MAGs was calculated (Fig. 4B). Thus, PAUC43f MAGs clustered according to their origin, in concordance with the 16S-based ecotaxonomy (Fig. 2 and 3). Indeed, the classification of 16S rRNA gene sequences retrieved from MAGs also showed that some genera were associated with specific environments (Fig. 2), supporting the specialization of these MAG lineages on specific ecological niches.

Both the phylogenomic tree and AAI values (Fig. 4B) indicated that the 52 MAGs represented 24 different species (AAI ≥ 95% (71, 79)), 10 of which were recovered at least twice from different metagenomes. MAGs from sponges belonged to 10 different species within the same genus, while the 14 species from saline soils and marine sediments fell into 8 different genera (AAI ≤ 65% (71)).

Based on these results and following the instructions of the recently published code for prokaryotes nomenclature from sequence data (SeqCode (80)), we propose renaming PAUC43f (=KS3-K002) as order *Palauibacterales*, in reference to the Republic of Palau, where the first 16S rRNA gene sequence of this group was retrieved. Thus, hereinafter, we will refer to PAUC43f as *Palauibacterales*. Additionally, we propose a name for the 7 genera and 16 species that meet the criteria of the SeqCode (Table 1; Table S3, SeqCode draft register list URL: seqco.de/r:0hkazsoc).

**TABLE 1:** Proposed taxonomic classification for *Palauibacterales* order^*a*^

Order	Family	Genera	Species	Species name
*Palauibacterales*	*Palauibacteraceae*	*Palauibacter*	Sp. 1	*Palauibacter polyketidifaciens*
			Sp. 2	*Palauibacter ramosifaciens*
			Sp. 3	*Palauibacter denitrificans*
			Sp. 4^T^	*Palauibacter soopunensis*
			Sp. 5	*Palauibacter irciniicola*
			Sp. 6	*Palauibacter rhopaloidicola*
			Sp. 7	*Palauibacter australiensis*
			Sp. 8	*Palauibacter scopulicola*
			Sp. 9	*Palauibacter poriticola*
		*Carthagonibacter*	Sp. 11	*Carthagonibacter metallireducens*
		*Benthicola*	Sp. 13^T^	*Benthicola marisminoris*
			Sp. 15	*Benthicola azotiphorus*
		*Humimonas*	Sp. 17^T^	*Humimonas hydrogenitrophica*
		*Caribbeanibacter*	Sp. 18^T^	*Caribbeanibacter nitroreducens*
		*Kutchimonas*	Sp. 22^T^	*Kutchimonas denitrificans*
		*Indicimonas*	Sp. 23^T^	*Indicimonas acetifermentans*

^*a*^ Protologue description can be found in Table S3.

### Core and niche-specific metabolic pathways in *Palauibacterales* MAGs

**Fig 5:**
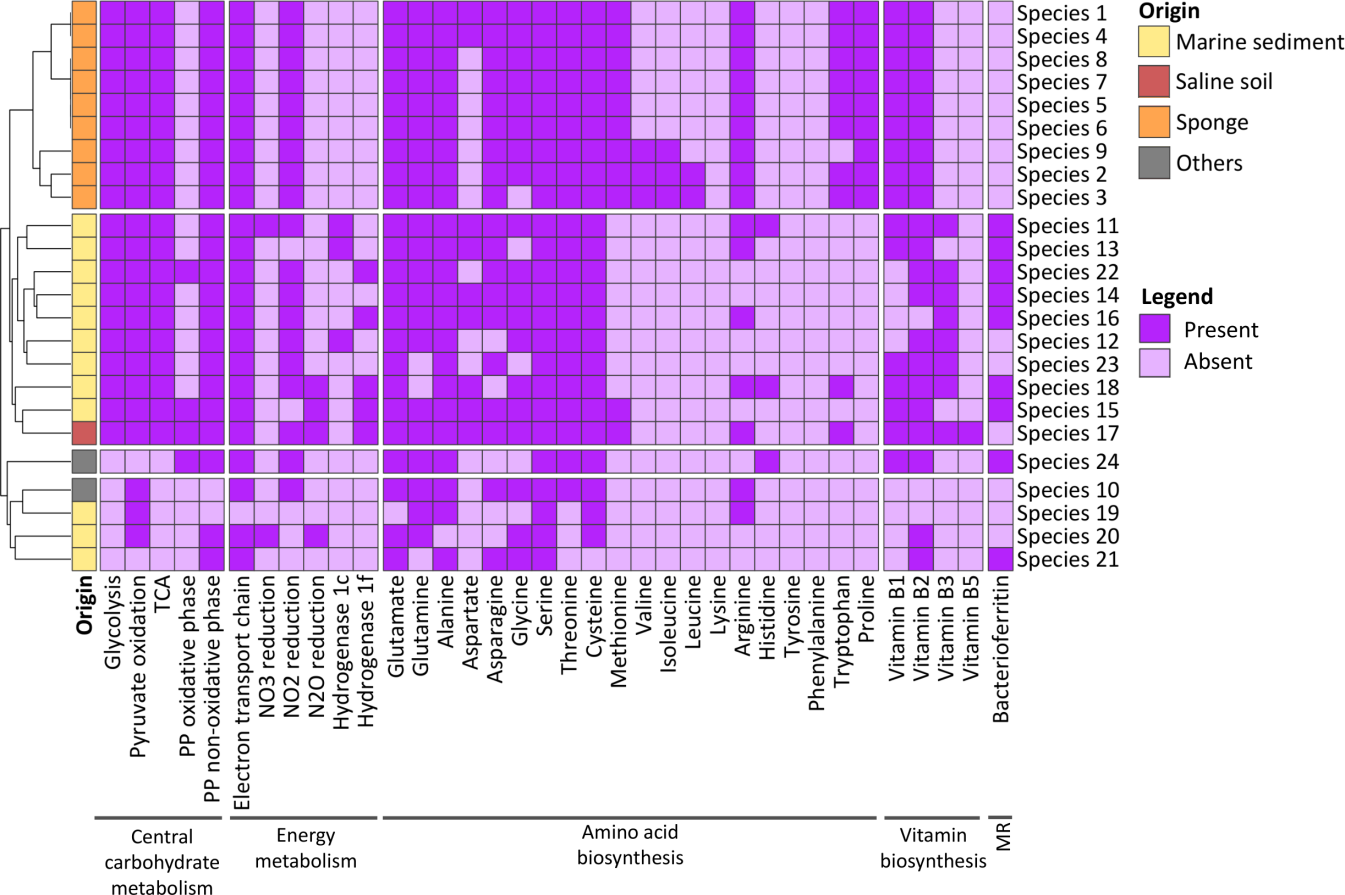
Heatmap showing the presence/absence of metabolic pathways within the 24 *Palauibacterales* species. A pathway was considered present if at least 80% of genes were detected. More detailed information can be found in Fig. S5 and Table S3. MR: Metal resistance.

To shed light on the ecological role of *Palauibacterales*, the potential metabolic capabilities of each species were explored (Fig. 5; Fig. S5; Table S3). MAG annotation indicated that *Palauibacterales* coded for typical gram-negative cell walls, as expected, and lacked the genes for flagella assembly (except species 18). Regarding central carbon metabolism, complete or almost complete glycolysis and tricarboxylic acid cycle (TCA) pathways were found in almost all species, as well as sugar transporters, pointing to *Palauibacterales* as a chemoorganotrophic bacteria. In good agreement, genes related to carbon fixation or photosynthetic metabolism were not found. However, species from sediments and saline soils presented 1 c and 1 f hydrogenases (81), so they may potentially shift between chemoorganotrophy and chemolithotrophy. It is worth noting that hydrogenotrophy has been recently demonstrated in other *Gemmatimonadota* members (82).

Members of the *Palauibacterales* are most likely facultative aerobes since genes for complex IV cytochrome oxidase, which transfers electrons to oxygen, were detected in almost all species. In addition, most of them also encoded genes for nitrate, nitrite, and/or nitrous oxide respiration, while the species retrieved from sponges were predicted to be able to respire thiosulfate, and species from sediments and saline soils might carry out acetate fermentation. The potential to reduce N_2_O by sediment and saline soils MAGs is in agreement with previous observations in other representatives of the phylum (8, 9, 83) and highlights *Palauibacterales* ecological relevance. Nitrous oxide is a potent greenhouse gas, which, due to human activities such as agricultural fertilization and combustion of fossil fuels (84), is increasing its atmospheric concentrations at a rate of 0.8 ppb per year (85), with some of the highest concentrations measured in coastal and estuarine waters (86, 87). Thus, N_2_O reducers, such as some *Palauibacterales* species may be, play a key role in mitigating the harmful effects of this gas. Furthermore, the *Palauibacterales* might have another restoring effect on the environment. In a recent study of Mar Menor (Spain) marine sediments, we observed a high relative abundance of PAUC43f in heavy-metal contaminated sediments (36). The most abundant PAUC43f OTU in these sediments shared 99.3% identity to the 16S rRNA gene sequence found in *Carthagonibacter metallireducens* (Sp. 11), which encodes for the hyaABCD NiFe hydrogenase that could act in hydrogenotrophic respirations using metals as electron acceptors, as previously described for *Geobacter sulfurreducens* (88). These observations suggest that some *Palauibacterales* species might obtain an ecological advantage by means of the respiration of metals, allowing them to thrive in these extreme environments. Furthermore, these species may be potential bioremediation agents in metal-contaminated areas.

With respect to amino acid biosynthesis, it is noteworthy that species from sponges were potentially able to synthesize more amino acids (12 - 15) than species from sediments and saline soils (5 - 12). The most common putative auxotrophies were found for lysine, tyrosine, phenylalanine, leucine, isoleucine, valine, and histidine. However, for some species these auxotrophies might be circumvented by acquiring amino acids from the environment using specific transporters (i.e., branched-chain amino acid transporters or transporters for oligopeptides). Since serine auxotrophy has been demonstrated for key marine microbes, such as *Pelagibacter ubique* (89), *Palauibacterales* may play a relevant ecological role in providing serine to the marine community.

Regarding the potential for vitamin B production, core biosynthetic genes for thiamine (vitamin B1) (*thiC*, *thiG,* and *thiE*), a cofactor of several essential enzymes (90), were detected in most species. Since B1 auxotrophy has been proposed as the second most common auxotrophy in marine environments (91), affecting both eukaryotes and prokaryotes (92 - 94), *Palauibacterales* might also be important suppliers of B1 to the marine communities. Genes for the complete biosynthetic operon of riboflavin (vitamin B2), a precursor of coenzymes FAD and FMN (95), and niacin (vitamin B3), a coenzyme in redox reactions, were also found in most species. The pathways for pantothenate (vitamin B5), a precursor of coenzyme A, and folate (vitamin B9), an important molecule in anabolic reactions, were partially present in these MAGs. If we assume that missing genes are a result of MAG incompleteness, *Palauibacterales* might also be capable of synthesizing these two vitamins. Biosynthetic pathways for vitamins B6, B7, and B12 were not found, and the presence of the *bioY* gene, which encodes a biotin (vitamin B7) transporter (96), and *btuF* and *btuB*, which are part of the cobalamin (vitamin B12) transporter (97), suggest that *Palauibacterales* may import these vitamins from the extracellular environment.

Secondary metabolites are usually involved in growth, development, and defense (98), and they are interesting molecules for medicine due to their potential uses as antibiotics, and antitumoral and cholesterol-lowering drugs. The search for BGCs with antiSMASH (67) revealed that sponge MAGs presented a higher number and diversity of BGCs (2, 9 BGCs per MAG) than those from sediments and saline soils (1, 2 BGCs per MAG) (Fig. S6A; Table S3). Despite the fact that most of the detected BGCs had no similarity to previously described BGCs, some T1PKS were similar to those known to synthetize azinomycin B, a potent antibiotic with antitumor activity (99, 100); cyphomycin, an antifungal compound (101); and vazabitide A and funisamine, both compounds with unknown biological properties (102, 103).

**Fig 6:**
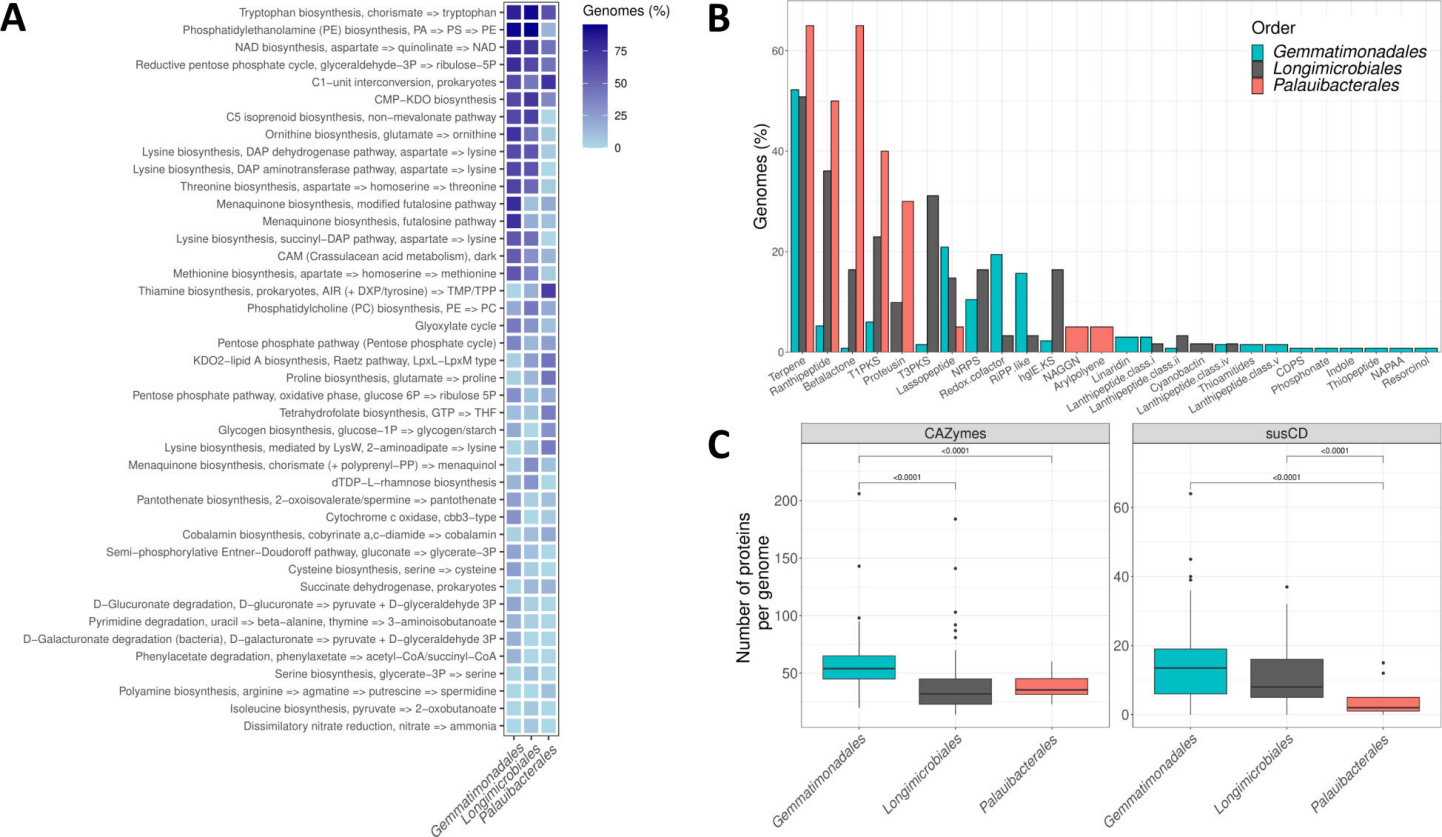
Differences in the potential metabolic and biosynthetic capabilities inferred from genomic data between the orders *Gemmatimonadales* (*n* = 134), *Longimicrobiales* (*n* = 61), and *Palauibacterales* (*n* = 20). (**A**) Frequency of KEGG modules with a minimum completeness of 80% displaying statistically significant differences between orders. Colors indicate the percentage of genomes/MAGs of each order that codify a given module. (**B**) Percentage of genomes/MAGs of each order that carry BGC. (**C**) Boxplot of the number of CAZymes and susCD proteins, markers of PULs, in the genomes/MAGs of each order. Only statistically significant differences (*P*-value < 0.05) reported by Wilcoxon test are shown.

CAZymes involved in biosynthesis, degradation, or modification of poly- and oligosaccharides showed clear differences in both abundance and composition between marine sediments, sponges, and saline soils MAGs (Fig. 6B and C). For example, families GH29 and GH95, both acting on fucose, a common polysaccharide in the marine environment, were detected only in marine sediment species. These differences in CAZymes composition might be related to the adaptation of *Palauibacterales* to the variety of niches they inhabit.

In addition, *Palauibacterales* MAGs encoded antibiotic resistance genes such as β-lactamases, tetracycline/H^+^ antiporters, and fosmidomycin and macrolide efflux pumps. Heavy metal resistance genes were also detected in sediment and saline soil species, including genes encoding efflux pumps for As^3+^, Zn^2+,^ and Fe^2+^and bacterioferritin, an iron storage protein which protects cells from reactive Fe^2+^.

### Order specific traits within the *Gemmatimonadetes* class

In an attempt to correlate the phylogeny with their metabolic potential, the main differences among the three largest *Gemmatimonadetes* orders (*Gemmatimonadales*, *Longimicrobiales,* and *Palauibacterales*) were explored. Notably, our results (Fig. 6) highlighted *Palauibacterales* as thiamine-producing bacteria, a trait with much less prevalence in the two other orders (Fig. 6A). This finding suggests either a higher limitation in the environment or a more relevant role of thiamine in *Palauibacterales* habitat. This vitamin is a coenzyme implicated in central metabolic processes such as the TCA cycle or the pentose-phosphate pathway and thus is essential for most living organisms (90). However, as mentioned above, previous studies have pointed to B1-auxotrophy as the second most common in the marine environment (91). About 25% of marine bacterial species require exogenous vitamin B1, a value that is notably higher in relevant marine taxa such as *Flavobacteriales* (76%) or *Rhodobacterales* (50%) (91). Furthermore, the number of B1 vitamin-requiring enzymes per genome is higher than for other vitamins, such as B7 or B12 (91), which is in agreement with the higher B1 uptake rates observed in coastal microbial communities (104). In addition (105), pointed out marine sediments, one of the main habitats of *Palauibacterales*, as sources of thiamine to the water column (105). Therefore, the literature highlights the relevance of B1-producing bacteria, such as *Palauibacterales* may be, in ecosystem functioning.

A second difference among the three orders was the presence in the *Palauibacterales* of BGC of ranthipeptide, betalactone, and proteusin, which were absent or less frequent in the other orders (Fig. 6B). Furthermore, polyketide synthase clusters were rare in *Gemmatimonadales*, whereas T1PKS were common in both *Palauibacterales* and *Longimicrobiales* and T3PKS in *Longimicrobiales*. These observations point to *Palauibacterales* as an interesting source of novel bioactive compounds with potential biotechnological applications.

Finally, the third main difference among orders has to do with the presence of polysaccharide utilization loci (PULs). These PULs are genomic *loci* that encode the necessary proteins to bind a given polysaccharide to the cell surface, cleaving it to oligosaccharides and importing them into the periplasmic region for their degradation to monosaccharides (106). They are typically composed of *susCD* genes, which transport the oligosaccharides from the extracellular to the periplasmic space, and CAZymes that catalyze polysaccharide degradation. Although PULs were thought to be restricted to *Bacteroidetes*, they were also observed in a few cultured genomes of *Gemmatimonadota* (106). Here, we show that PULs are widely distributed within this phylum and are not a rarity, as previously believed (Fig. 6C Fig. S7). The prevalence of PULs (based on the number of CAZymes and susCD genes) is significantly higher in the genomes of *Gemmatimonadales* and *Longimicrobiales* than in *Palauibacterales* (Fig. 6C). This observation might indicate that *Palauibacterales* present less potential for importing and degrading polysaccharides than its sister orders within the phylum.

**Fig 7:**
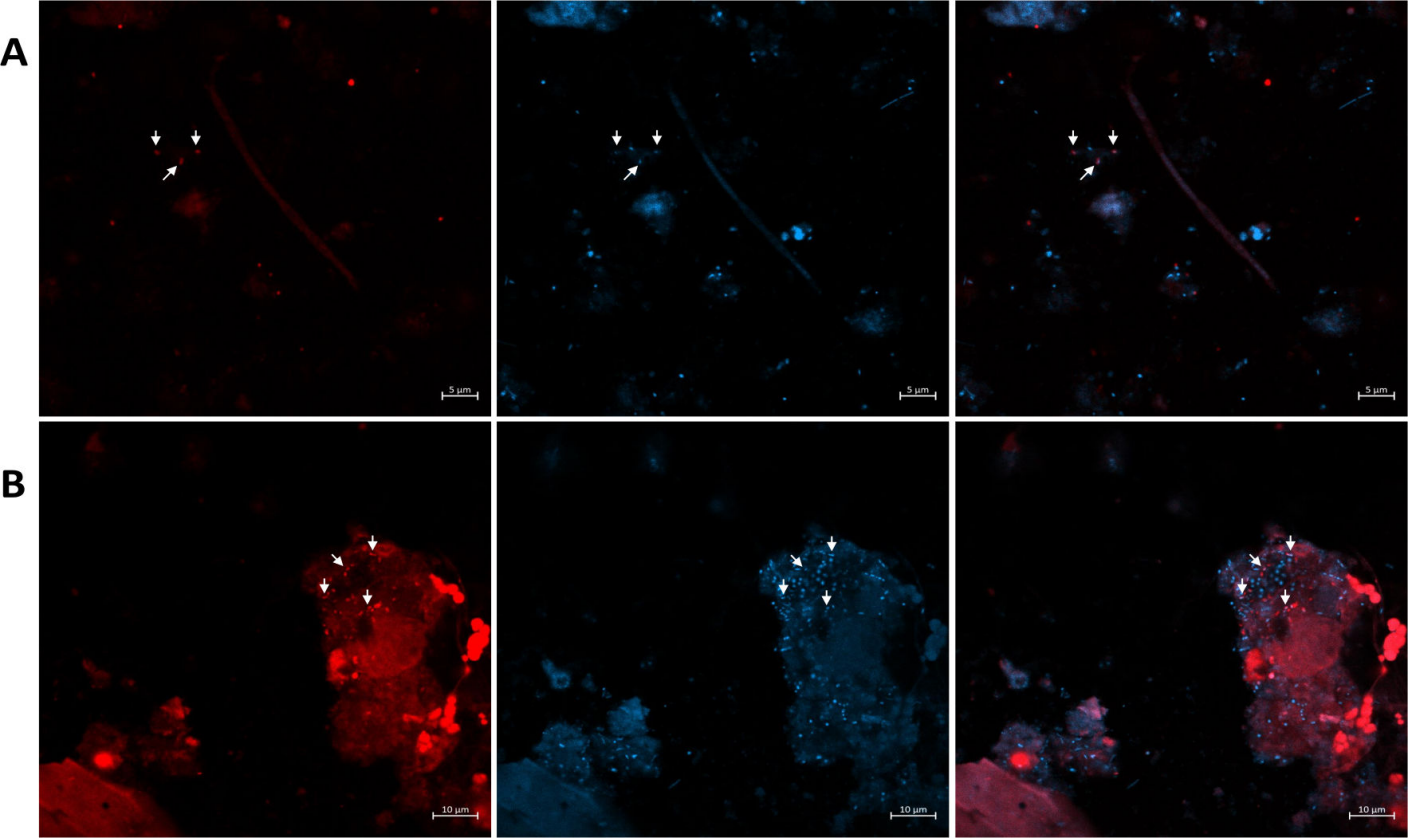
FISH of Mar Menor sediment samples with a *Palauibacterales* specific probe. A and B show two different microscopic fields observed with two different color channels, red for probe and blue for DAPI. From left to right: PAUC43f_826, DAPI, and merged channels. Arrows indicate cells displaying signals in both FISH and DAPI channels.

### Visualization of active *Palauibacterales* in marine sediments

To visualize *Palauibacterales* cells and evaluate their metabolically active state in the environment, a FISH probe was designed and tested. *In silico* analyses indicated that the probe matched 32% of the *Palauibacterales* sequences deposited in the SILVA database and sequences of genera 2, 3, 4, 5, 6, 7, and 8 (Fig. 2). Thus, the probe does not target the whole *Palauibacterales* order but rather a set of closely related sequences, mostly associated with marine sediments and saline soils. Since the probe also matched 16S rRNA gene sequences from MAGs recovered from Mar Menor sediments, FISH was performed with sediment samples from this lagoon. The best hybridization was obtained with 40% formamide and, as shown in Fig. 7, *Palauibacterales* cells displayed a small but wide rod morphology. Considering that the number of hybridized cells seemed to be higher than the cells stained with DAPI, we suspected DAPI might have been quenched by the probe fluorophore or by pigments present in the cells. With this assay, we provide experimental evidence of the presence and metabolically active state of the order *Palauibacterales* in marine sediments.

### Final remarks

Based on the ubiquity of the *Gemmatimonadota* phylum in the marine environment, Hanada and Sekiguchi noted that this phylum may play an important but still unknown ecological role (4). The results presented here highlight the ecological relevance of a key unexplored order in that phylum, the *Palauibacterales*, within marine environments. This cosmopolitan order within the *Gemmatimonadetes* class displays a salt-related character and presents interesting potential metabolic features, such as N_2_O reduction and serine and thiamine biosynthesis, with the latter as a probable key trait of the group. The presence of PULs in most *Gemmatimonadetes* expands the capability for complex polysaccharide degradation beyond the well-known *Bacteroidetes* and *Verrucomicrobia*. With this work, we provide evidence that the influence of *Gemmatimonadota* on marine ecosystem functioning, despite having been overlooked to date, may be more significant than previously supposed.

## Data Availability

The list of SRA 16S rRNA gene amplicon runs used in this study are available in Table S1. The accession numbers for the MAGs employed in this study can be found in Table S3.

## SUPPLEMENTAL MATERIAL

Fig S1 msystems.00215-23-s0001.pdf PAUC43f abundance based on 16S rRNA gene sequences in sediments as functions of A) latitude, B) temperature, C) water column depth above the sediment, and D) sediment depth.

Fig S2 msystems.00215-23-s0002.pdf PAUC43f abundance based on 16S rRNA gene sequences in soils as function of A) latitude and B) soil depth.

Fig S3 msystems.00215-23-s0003.pdf Dotplot between the mean abundance (% of 16S rRNA reads) and the number of samples per depth bin for sediments (A) and soils (B).

Fig S4 msystems.00215-23-s0004.pdf Boxplot of the A) estimated MAG size and B) completeness related to the environment from which they were recovered. Statistically significant p-values reported by ANOVA are shown above boxplots.

Fig S5 msystems.00215-23-s0005.pdf Predicted metabolic capabilities for the 24 *Palauibacterales* species. Each species is represented by a colored dot (see legend) named in the same order as in Figure 4. The annotation of MAGs used to reconstruct the metabolism can be found in Supplementary Table 4.

Fig S6 msystems.00215-23-s0006.pdf Differences within the *Palauibacterales* order with regard to the MAG origin. A) Secondary metabolite biosynthetic gene clusters (BGCs) predicted by antiSMASH for each MAG. The colored background of species’ names shows the origin of the species (green: marine sediment; red: saline soils; and orange: sponges) and colored dots indicate the number of each BGC per MAG (1: blue; 2: yellow; 3: orange; and 4: red). B) Boxplot of the number of annotated CAZymes per genome. C) NMDS plot based on Bray-Curtis distances calculated from a matrix of CAZymes composition and abundance in each genome.

Fig S7 msystems.00215-23-s0007.pdf Examples of PUL in the three orders of *Gemmatimonadota*. Colored dots at the left indicate the order, following the same color schema as in previous figures. PUL sequences were identified from MAG GCA_016713785.1 for *Gemmatimonadales*, 3300025924_14 for *Longimicrobiales,* and Bin_S212_14 for *Palauibacterales*.

Table S1 msystems.00215-23-s0008.xlsx Metadata of the 16S rRNA gene amplicon datasets where PAUC43f was detected.

Table S2 msystems.00215-23-s0009.xlsx First worksheet, pairwise Wilcoxon test between the abundance of PAUC43f in different environments (see Figure 1). P-values were corrected by Bonferroni. Only groups with more than 16 samples were tested to avoid biased results due to small sample size. Second and following worksheets, pairwise Wilcoxon test for the abundance of each PAUC43f genus in different environments (see Figure 3). P-values were corrected by Bonferroni. Only groups with more than 16 samples were tested to avoid biased results due to small sample size.

Table S3 msystems.00215-23-s0010.xlsx First worksheet, general characteristics of *Palauibacterales* MAGs. aStrain heterogeneity. bMAG abundance is shown as a percentage of recruited reads from the total metagenome reads. CMAG normalized abundance is shown as the number of recruited reads divided by metagenome and genome size. Second worksheet, protologue for the new described taxa within the *Palauibacterales* order. Third and following worksheets, annotation of predicted proteins from MAGs using the KEGG KO, Pfam, CDD, SMART, TIGRFAM, and AntiSMASH.

ASM does not own the copyrights to Supplemental Material that may be linked to, or accessed through, an article. The authors have granted ASM a non-exclusive, world-wide license to publish the Supplemental Material files. Please contact the corresponding author directly for reuse.
