# Supplementary material for: Distribution, abundance, and ecogenomics of the Palauibacterales, a new cosmopolitan thiamine-producing order within the Gemmatimonadota phylum
Source: mSystems. 2023 Jun 22;8(4):e00215-23. doi: 10.1128/msystems.00215-23 (PMC10469786; doi:10.1128/msystems.00215-23)
Supplement: Fig S1 — PAUC43f abundance based on 16S rRNA gene sequences in sediments as functions of A) latitude, B) temperature, C) water column depth above the sediment, and D) sediment depth. [file msystems.00215-23-s0001.pdf]

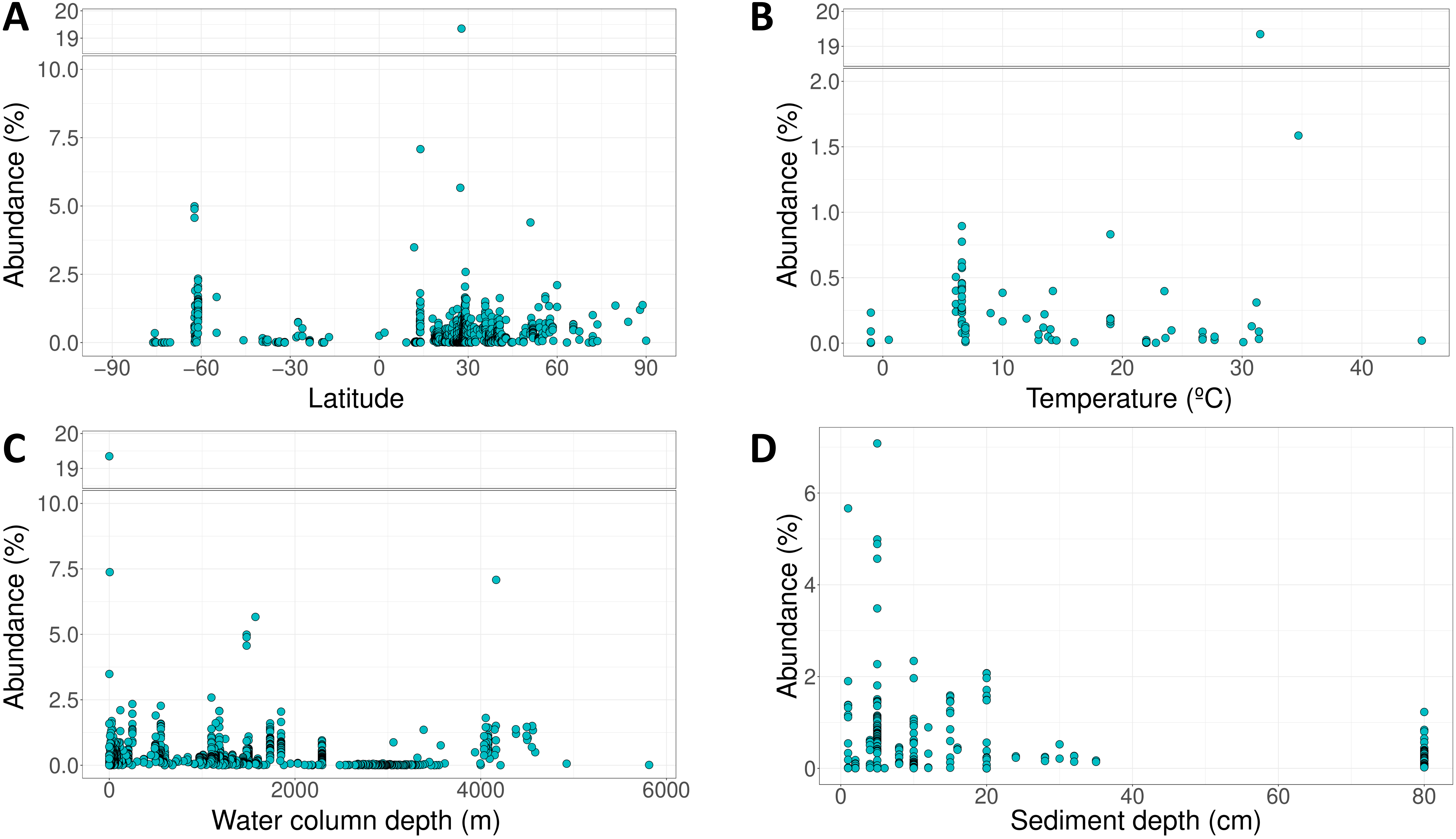

Supplementary Figure 1. PAUC43f abundance based on 16S rRNA gene sequences in sediments as function of A) latitude, B) temperature, C) water column depth above the sediment and D) sediment depth.
