# Supplementary material for: Distribution, abundance, and ecogenomics of the Palauibacterales, a new cosmopolitan thiamine-producing order within the Gemmatimonadota phylum
Source: mSystems. 2023 Jun 22;8(4):e00215-23. doi: 10.1128/msystems.00215-23 (PMC10469786; doi:10.1128/msystems.00215-23)
Supplement: Fig S2 — PAUC43f abundance based on 16S rRNA gene sequences in soils as function of A) latitude and B) soil depth. [file msystems.00215-23-s0002.pdf]

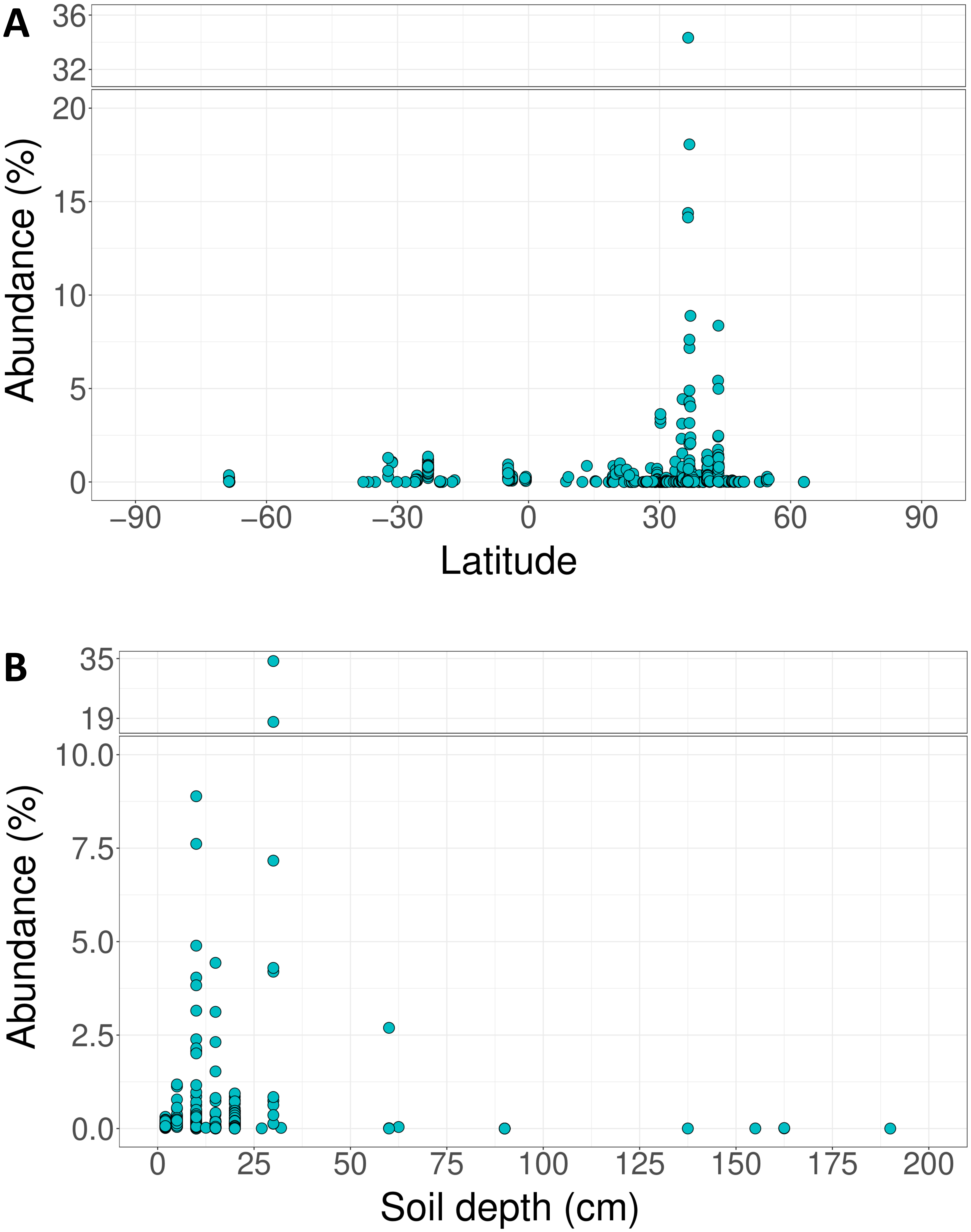

Supplementary Figure 2. PAUC43f abundance based on 16S rRNA gene sequences in soils as function of A) latitude and B) soil depth.
