# Supplementary material for: Distribution, abundance, and ecogenomics of the Palauibacterales, a new cosmopolitan thiamine-producing order within the Gemmatimonadota phylum
Source: mSystems. 2023 Jun 22;8(4):e00215-23. doi: 10.1128/msystems.00215-23 (PMC10469786; doi:10.1128/msystems.00215-23)
Supplement: Fig S3 — Dotplot between the mean abundance (% of 16S rRNA reads) and the number of samples per depth bin for sediments (A) and soils (B). [file msystems.00215-23-s0003.pdf]

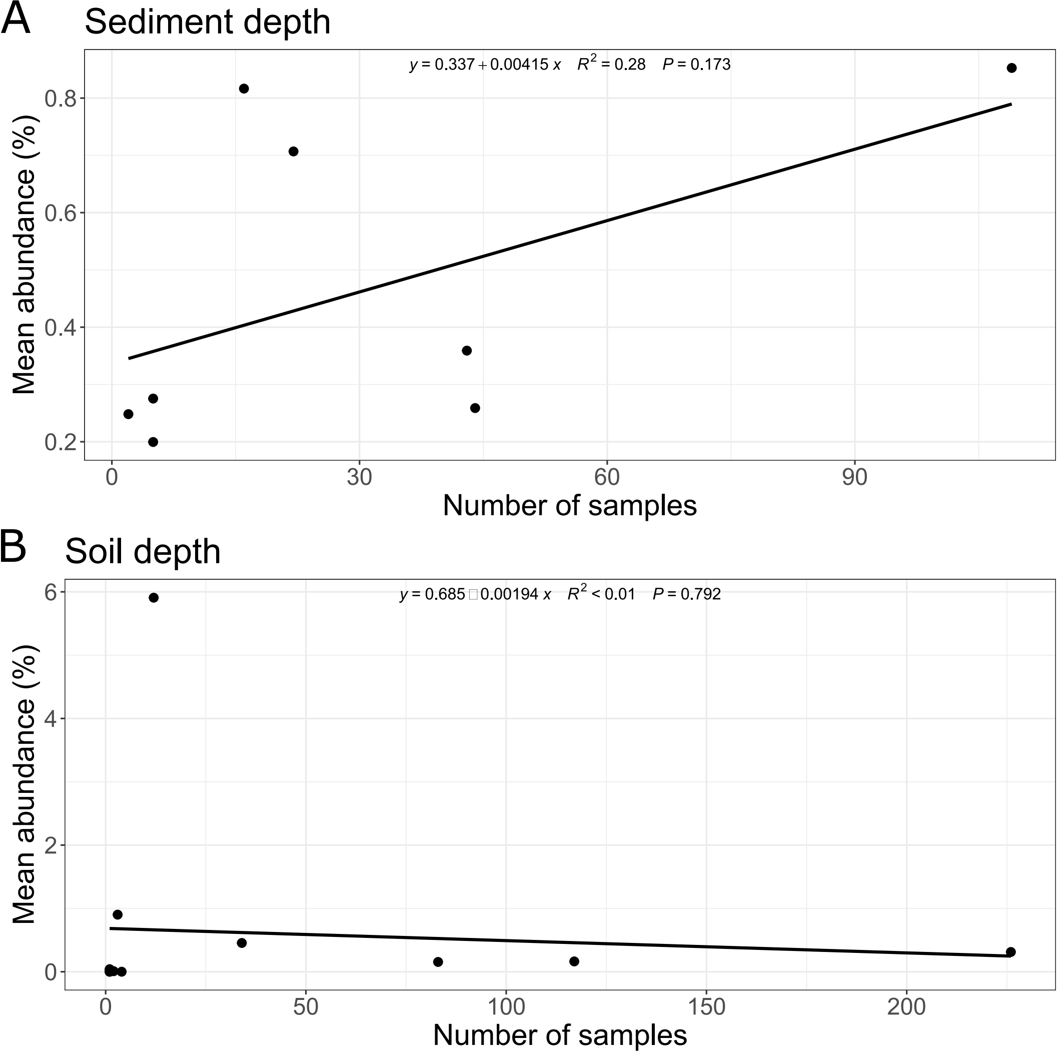

Supplementary Figure 3. Dotplot between the mean abundance (% of 16S rRNA reads) and the number of samples per depth bin for sediments (A) and soils (B).
