# Supplementary material for: Distribution, abundance, and ecogenomics of the Palauibacterales, a new cosmopolitan thiamine-producing order within the Gemmatimonadota phylum
Source: mSystems. 2023 Jun 22;8(4):e00215-23. doi: 10.1128/msystems.00215-23 (PMC10469786; doi:10.1128/msystems.00215-23)
Supplement: Fig S4 — Boxplot of the A) estimated MAG size and B) completeness related to the environment from which they were recovered. Statistically significant p-values reported by ANOVA are shown above boxplots. [file msystems.00215-23-s0004.pdf]

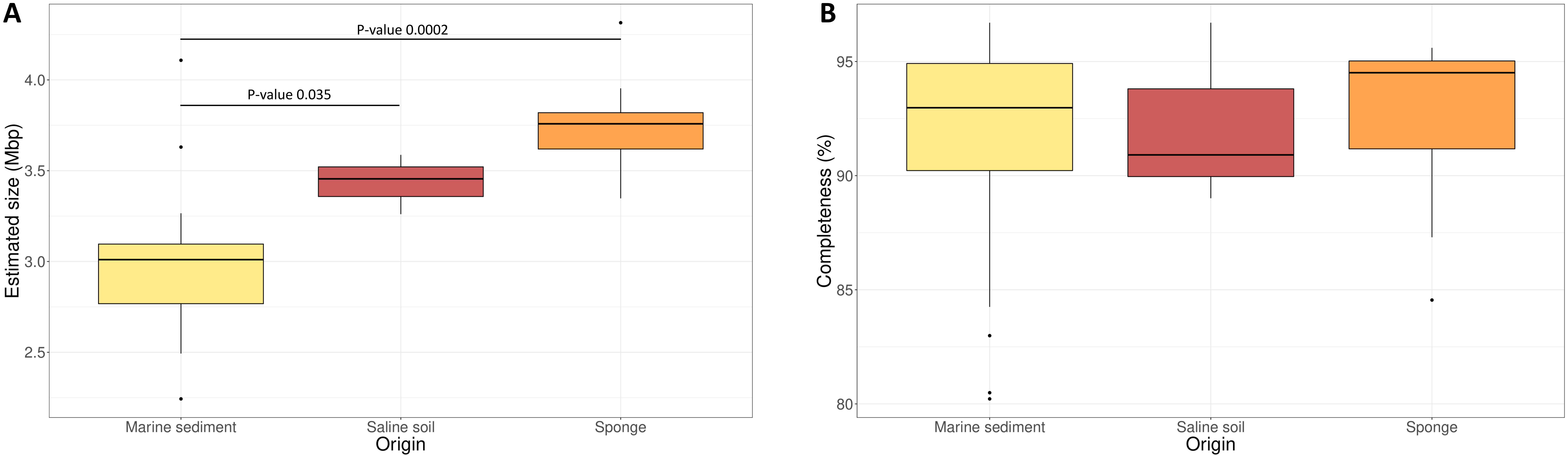

Supplementary Figure 4. Boxplot of the A) estimated MAG size and B) completeness related to the environment from which they were recovered. Statistically significant p-values reported by ANOVA are shown above boxplots.
