# Supplementary material for: Distribution, abundance, and ecogenomics of the Palauibacterales, a new cosmopolitan thiamine-producing order within the Gemmatimonadota phylum
Source: mSystems. 2023 Jun 22;8(4):e00215-23. doi: 10.1128/msystems.00215-23 (PMC10469786; doi:10.1128/msystems.00215-23)
Supplement: Fig S5 — Predicted metabolic capabilities for the 24 Palauibacterales species. Each species is represented by a colored dot (see legend) named in the same order as in Figure 4. The annotation of MAGs used to reconstruct the metabolism can be found in Supplementary Table 4. [file msystems.00215-23-s0005.pdf]

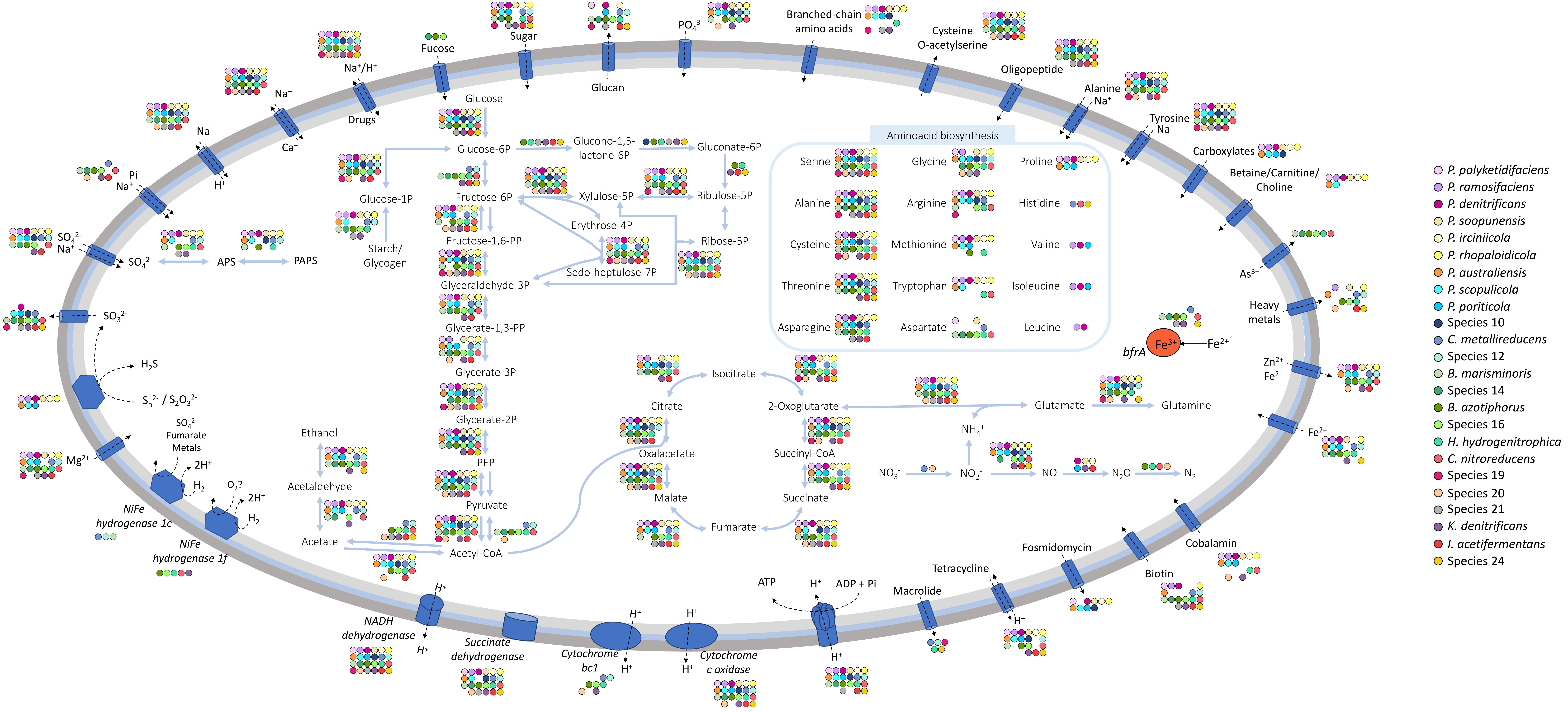

Supplementary Figure 5. Predicted metabolic capabilities for the 24 *Palauibacterales* species. Each species is represented by a colored dot (see legend) named in the same order as in Figure 4. The annotation of MAGs used to reconstruct the metabolism can be found in Supplementary Table 4.
