# Supplementary material for: Distribution, abundance, and ecogenomics of the Palauibacterales, a new cosmopolitan thiamine-producing order within the Gemmatimonadota phylum
Source: mSystems. 2023 Jun 22;8(4):e00215-23. doi: 10.1128/msystems.00215-23 (PMC10469786; doi:10.1128/msystems.00215-23)
Supplement: Fig S6 — Differences within the Palauibacterales order with regard to the MAG origin. A) Secondary metabolite biosynthetic gene clusters (BGCs) predicted by antiSMASH for each MAG. The colored background of species’ names shows the origin of the species (green: marine sediment; red: saline soils; and orange: sponges) and colored dots indicate the number of each BGC per MAG (1: blue; 2: yellow; 3: orange; and 4: red). B) Boxplot of the number of annotated CAZymes per genome. C) NMDS plot based on Bray-Curtis distances calculated from a matrix of CAZymes composition and abundance in each genome. [file msystems.00215-23-s0006.pdf]

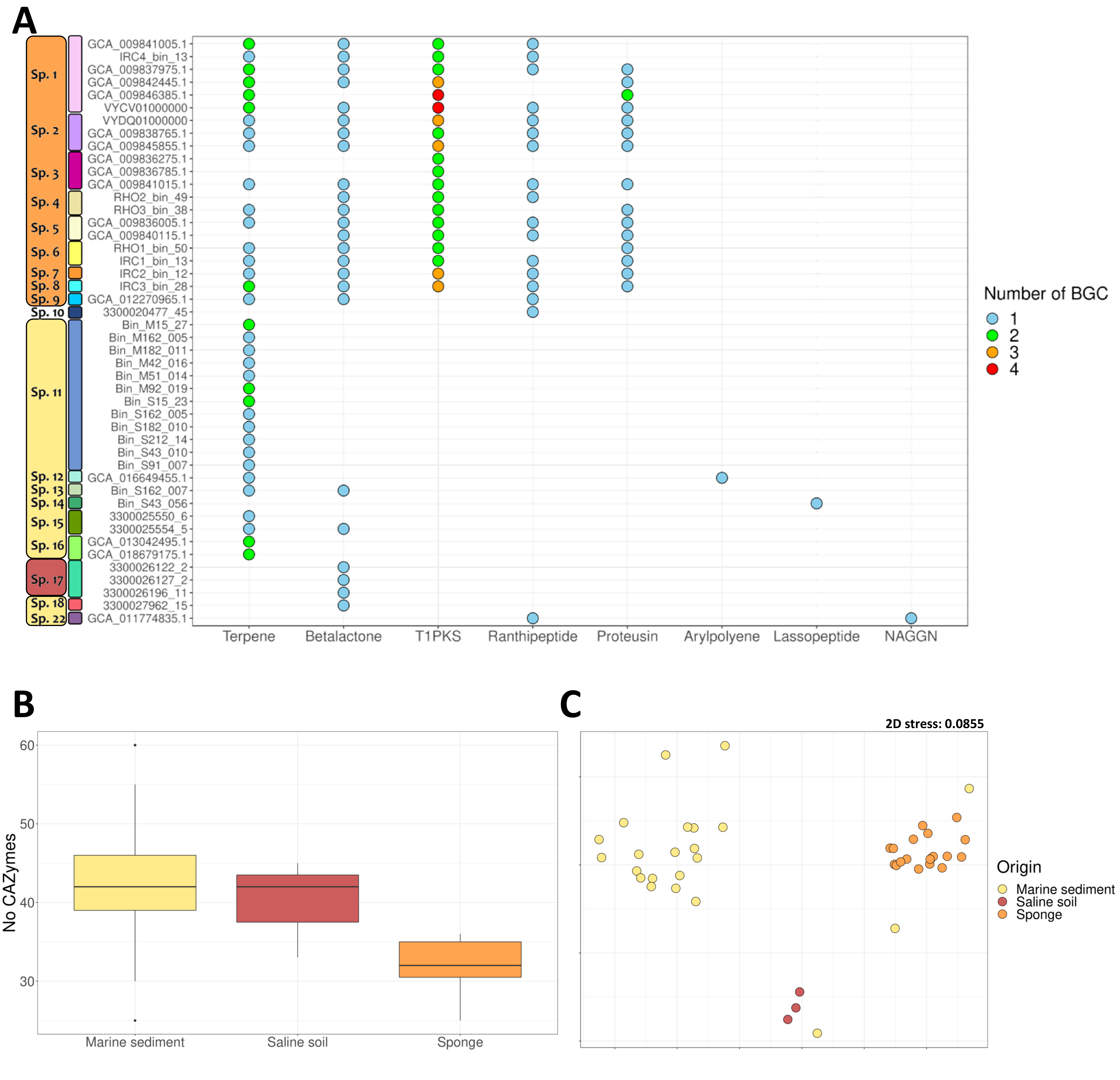

Supplementary Figure 6. Differences within the *Palauibacterales* order in regards of the MAG origin. A) Secondary metabolite biosynthetic gene clusters (BGC) predicted by antiSMASH for each MAG. The colored background of species names shows the origin of the species (green: marine sediment; red: saline soils; orange: sponges) and colored dots indicate the number of each BGC per MAG (1: blue; 2: yellow; 3: orange; 4: red). B) Boxplot of the number of annotated CAZymes per genome. C) NMDS plot based on Bray-Curtis distances calculated from a matrix of CAZymes composition and abundance in each genome.
