# Supplementary material for: Distribution, abundance, and ecogenomics of the Palauibacterales, a new cosmopolitan thiamine-producing order within the Gemmatimonadota phylum
Source: mSystems. 2023 Jun 22;8(4):e00215-23. doi: 10.1128/msystems.00215-23 (PMC10469786; doi:10.1128/msystems.00215-23)
Supplement: Fig S7 — Examples of PUL in the three orders of Gemmatimonadota. Colored dots at the left indicate the order, following the same color schema as in previous figures. PUL sequences were identified from MAG GCA_016713785.1 for Gemmatimonadales, 3300025924_14 for Longimicrobiales, and Bin_S212_14 for Palauibacterales. [file msystems.00215-23-s0007.pdf]

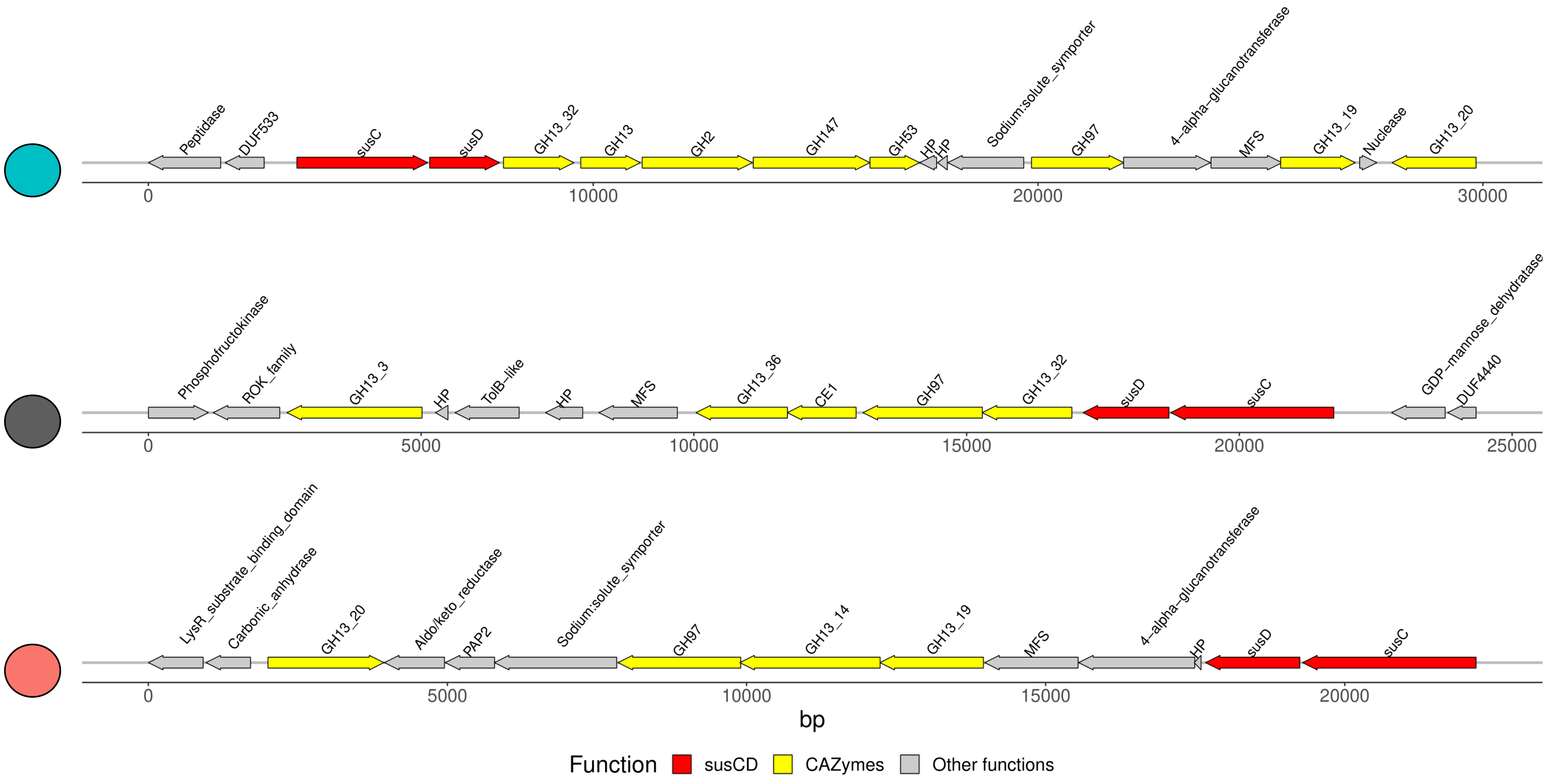

Supplementary Figure 7. Examples of PUL in the three orders of *Gemmatimonadota*. Colored dots at the left indicate the order, following the same color schema as in previous figures. PUL sequences were identified from MAG GCA_016713785.1 for *Gemmatimonadales*, 3300025924_14 for *Longimicrobiales* and Bin_S212_14 for *Palauibacterales*.
